# Supplementary material for: Trends of inequality in DPT3 immunization services utilization in Ethiopia and its determinant factors: Evidence from Ethiopian demographic and health surveys, 2000–2019
Source: PLoS One. 2024 Jan 16;19(1):e0293337. doi: 10.1371/journal.pone.0293337 (PMC10791004; doi:10.1371/journal.pone.0293337)
Supplement: S1 Table — (DOCX) [file pone.0293337.s001.docx]

**Table 4: Community and individual level factors associated with DPT3 immunization using Multilevel Binary logistic Regression in under –five children from 2000 to 2019.**

| *Predictors* |  | *Odds Ratios* | *CI* | *p-value* |
| --- | --- | --- | --- | --- |
|  | Intercept | 0.40 | 0.28 – 0.57 | **<0.001** |
| Community-level variables | | | | |
| Survey Year | 2005 | 1.48 | 1.26 – 1.75 | **<0.001** |
|  | 2011 | 1.79 | 1.52 – 2.11 | **<0.001** |
|  | 2016 | 2.93 | 2.46 – 3.48 | **<0.001** |
|  | 2019 | 3.15 | 2.56 – 3.89 | **<0.001** |
| Region | Tigray | 1.29 | 0.93 – 1.79 | 0.124 |
|  | Afar | 0.05 | 0.03 – 0.09 | **<0.001** |
|  | Amhara | 0.46 | 0.34 – 0.63 | **<0.001** |
|  | Oromia | 0.26 | 0.19 – 0.35 | **<0.001** |
|  | Somali | 0.09 | 0.06 – 0.14 | **<0.001** |
|  | Ben Gumz | 0.35 | 0.23 – 0.52 | **<0.001** |
|  | SNNP | 0.31 | 0.23 – 0.43 | **<0.001** |
|  | Gambella | 0.17 | 0.09 – 0.31 | **<0.001** |
|  | Harari | 0.43 | 0.23 – 0.80 | **0.008** |
|  | Dire Dawa | 0.89 | 0.52 – 1.53 | 0.683 |
| Residence | Rural | 0.50 | 0.42 – 0.60 | **<0.001** |
| Individual-level variables | | | | |
| Wealth | Poorer | 0.98 | 0.89 – 1.08 | 0.678 |
|  | Middle | 1.05 | 0.95 – 1.17 | 0.317 |
|  | Richer | 1.26 | 1.13 – 1.40 | **<0.001** |
|  | Richest | 1.21 | 1.04 – 1.41 | **0.013** |
| Education | Primary | 1.19 | 1.10 – 1.29 | **<0.001** |
|  | Secondary/Higher | 1.25 | 1.08 – 1.44 | **0.002** |
| Sex of child | Female | 0.90 | 0.84 – 0.95 | **<0.001** |
| Birth order | Second | 0.98 | 0.88 – 1.09 | 0.701 |
|  | Third | 0.97 | 0.87 – 1.09 | 0.609 |
|  | Forth or above | 0.83 | 0.74 – 0.93 | **0.001** |
| Age of respondent | Years | 1.02 | 1.02 – 1.03 | **<0.001** |
| ANC | Two | 1.06 | 0.91 – 1.22 | 0.457 |
|  | Three | 1.67 | 1.48 – 1.89 | **<0.001** |
|  | Four or more | 2.58 | 2.39 – 2.79 | **<0.001** |
| Place of delivery | Health facility | 1.18 | 1.07 – 1.31 | **0.001** |
| Media | Yes | 0.98 | 0.91 – 1.06 | 0.698 |
| **Random Effects** | | | | |
|  | σ^2^ | 3.29 | | |
|  | τ_00_ _cluster_ | 0.75 | | |
|  | ICC | 0.19 | | |

*The ICC (Intra class correlation), 19%, suggest the necessity of multilevel modeling.

*Reference categories of community-level variable: EDHS 2000, Addis Ababa, urban

*Reference categories of individual-level variables: Poorest, no education, male, first, one
