# Supplementary material for: Trends of inequality in DPT3 immunization services utilization in Ethiopia and its determinant factors: Evidence from Ethiopian demographic and health surveys, 2000–2019
Source: PLoS One. 2024 Jan 16;19(1):e0293337. doi: 10.1371/journal.pone.0293337 (PMC10791004; doi:10.1371/journal.pone.0293337)
Supplement: Attachment — Submitted filename: Review comments_Dr S Sarkar.pdf [file pone.0293337.s003.pdf]

Review Comments

Title: Trends of Inequality in DPT3 Immunization Services Utilization in Ethiopia and its Determinant factors: Evidence from Ethiopian Demographic and Health Surveys, 2000-2019

Thank you for allowing me to review a manuscript entitled "Trends of Inequality in DPT3 Immunization Services Utilization in Ethiopia and its Determinant factors: Evidence from Ethiopian Demographic and Health Surveys, 2000- 2019" for your esteemed journal. However, after a careful review, I find the following points to share with you:

1. The introduction section is very general. The author may include a global overview of immunization, referring to developed and developing countries.
2. The literature review should also include the major challenges in utilizing immunization across the globe.
3. Research gap needs to be strengthened.
4. The study has used historical data from 2000 till 2019; therefore, it is important to mention the sample size and data compatibility across the various rounds.
5. The methods of inequality used in the study are very general and require methodological definitions for better clarification. Moreover, authors may think to use some advanced methods of inequality in spite of multiple methods, such as the Gini index etc.,
6. There should have been a sample summary table to understand the background characteristics of the sample of various rounds.
7. Although the higher wealth index group shows higher coverage in the utilization compared to the poorest group, the interpretation should also look into the fact that the level of utilization has increased at three times during the mentioned period among the poorest group, which is much higher than the highest quintile group.
8. There is a significant increase in the level of immunization among poor people, the authors should discuss the policy efforts and other dynamics for such improvement in the discussion section.

Reccomondadtion: This is an important study in the field but requires major revisions.

Dr Sanjit Sarkar

***
