# Supplementary material for: Trends of inequality in DPT3 immunization services utilization in Ethiopia and its determinant factors: Evidence from Ethiopian demographic and health surveys, 2000–2019
Source: PLoS One. 2024 Jan 16;19(1):e0293337. doi: 10.1371/journal.pone.0293337 (PMC10791004; doi:10.1371/journal.pone.0293337)
Supplement: Attachment — Submitted filename: Response to Reviews.docx [file pone.0293337.s004.docx]

**Response to Review Comments**

Title: Trends of Inequality in DPT3 Immunization Services Utilization in Ethiopia and its Determinant factors: Evidence from Ethiopian Demographic and Health Surveys, 2000- 2019 Thank you for allowing me to review a manuscript entitled "Trends of Inequality in DPT3 Immunization Services Utilization in Ethiopia and its Determinant factors: Evidence from Ethiopian Demographic and Health Surveys, 2000- 2019" for your esteemed journal. However, after a careful review, I find the following points to share with you:

1. The introduction section is very general. The author may include a global overview of immunization, referring to developed and developing countries.

#Response: Yes you are right, now it is updated and corrected based on your valuable comment

2. The literature review should also include the major challenges in utilizing immunization across the globe.

#Response: Yes you are correct, now it is updated and major challenges in utilizing immunization across the globe is included based on your valuable comment

3. Research gap needs to be strengthened.

#Response: Yes you are right, now it is updated and research gaps needs to be strengthen were included based on your valuable comment

4. The study has used historical data from 2000 till 2019; therefore, it is important to mention the sample size and data compatibility across the various rounds.

#Response: Yes you are right, now the sample size and data compatibility across the various rounds was mentioned in the manuscript based on your valuable comment

5. The methods of inequality used in the study are very general and require methodological definitions for better clarification. Moreover, authors may think to use some advanced methods of inequality in spite of multiple methods, such as the Gini index etc.

#Response: Yes you are right, now methodological definitions were given based on your valuable comment and some advanced methods of data analysis have been done and shown in the manuscript and concentration curve is attached as Fig 7 with this revised manuscript

6. There should have been a sample summary table to understand the background characteristics of the sample of various rounds.

#Response: Yes you are right, now sample summary table has been included in the manuscript to show and to understand characteristics of the samples as indicated in your valuable comment

7. Although the higher wealth index group shows higher coverage in the utilization compared to the poorest group, the interpretation should also look into the fact that the level of utilization has increased at three times during the mentioned period among the poorest group, which is much higher than the highest quintile group.

#Response: Yes it is true, now it is updated and corrected based on your valuable comment

8. There is a significant increase in the level of immunization among poor people, the authors should discuss the policy efforts and other dynamics for such improvement in the discussion section.

#Response: Yes you are right, now it is updated and some justification is given based on your valuable comment

9. Recommendation: This is an important study in the field but requires major revisions.

Dr Sanjit Sarkar

Author : I thank you for your detail reviewing and your valuable comment
