# Supplementary material for: Trends of inequality in DPT3 immunization services utilization in Ethiopia and its determinant factors: Evidence from Ethiopian demographic and health surveys, 2000–2019
Source: PLoS One. 2024 Jan 16;19(1):e0293337. doi: 10.1371/journal.pone.0293337 (PMC10791004; doi:10.1371/journal.pone.0293337)
Supplement: Attachment — Submitted filename: Comments on Revised paper.docx [file pone.0293337.s005.docx]

Review comments

**Title of the paper**: Trends of Inequality in DPT3 Immunization Services Utilization in Ethiopia and its Determinant factors: Evidence from Ethiopian Demographic and Health Surveys, 2000- 2019

Comments:

Thank you for the opportunity to review the revised version of the paper. The authors addressed all the quarries, clarifications, and changes raised in a very systematic manner. I congratulate all the authors for writing this manuscript and recommend that the journal editor consider the manuscript for publication after verifying the proofreading.
